# Physiological functions of the effects of the different bathing method on recovery from local muscle fatigue

**DOI:** 10.1186/1880-6805-31-26

**Published:** 2012-09-16

**Authors:** Soomin Lee, Shogo Ishibashi, Yoshihiro Shimomura, Tetsuo Katsuura

**Affiliations:** 1 Center for Environment, Health and Field Sciences, Chiba University, Chiba, Japan; 2 Graduate School of Engineering, Chiba University, Chiba, Japan

**Keywords:** Mist sauna, Full immersion bath, Muscle fatigue, EMG, SBF, O2Hb

## Abstract

**Background:**

Recently, mist saunas have been used in the home as a new bathing style in Japan. However, there are still few reports on the effects of bathing methods on recovery from muscle fatigue. Furthermore, the effect of mist sauna bathing on human physiological function has not yet been revealed. Therefore, we measured the physiological effects of bathing methods including the mist sauna on recovery from muscle fatigue.

**Methods:**

The bathing methods studied included four conditions: full immersion bath, shower, mist sauna, and no bathing as a control. Ten men participated in this study. The participants completed four consecutive sessions: a 30-min rest period, a 10-min all out elbow flexion task period, a 10-min bathing period, and a 10-min recovery period. We evaluated the mean power frequency (MNF) of the electromyogram (EMG), rectal temperature (Tre), skin temperature (Tsk), skin blood flow (SBF), concentration of oxygenated hemoglobin (O2Hb), and subjective evaluation.

**Results:**

We found that the MNF under the full immersion bath condition was significantly higher than those under the other conditions. Furthermore, Tre, SBF, and O2Hb under the full immersion bath condition were significantly higher than under the other conditions.

**Conclusions:**

Following the results for the full immersion bath condition, the SBF and O2Hb of the mist sauna condition were significantly higher than those for the shower and no bathing conditions. These results suggest that full immersion bath and mist sauna are effective in facilitating recovery from muscle fatigue.

## Background

Bathing is a custom that is deeply ingrained in the life of Japanese people [1]. Unlike their European and American counterparts [2], many Japanese are thought to bathe in a bathtub almost daily [3]. Bathing, or soaking, in a bathtub is a popular and often habitual pastime that has its roots in the culture of the Japanese people [4]. In general, it is well-known that Japanese people prefer bathing for recovery from fatigue as well as to ensure cleanliness [5].

Based on the early studies, fatigue has been defined as the difficulty in initiating or sustaining voluntary activities [6], is a common symptom of various illnesses, and could even be observed in healthy individuals [7-9].

On the other hand, Mizuno *et al.* reported that fatigue could be classified as acute or chronic fatigue [10]. In order to avoid chronic fatigue, it is important to develop effective strategies to recover to avoid the accumulation of acute fatigue.

Regarding the style of bathing, it was reported that heart rate was higher, muscle stiffness in the waist was lower, and plasma cortisol levels tended to be lower after mild-stream bathing than after a full immersion bath [10]. Johnston *et al*. showed that higher oxygen consumption and electrocardiographic changes were found during showering than during a full immersion bath [11]. By contrast, it was reported that muscle performance related to muscle temperature as follows: maximal isometric force production, the rates of force development and relaxation [12-15], and maximal power production (Pmax) [16-19] decrease with a decrease in muscle temperature. Recently, various bathing methods have become popular in Japan, including full immersion bath, showering, and so on. Among them, the mist sauna has emerged as a new home-bathing method. The mist sauna is well-known as a low-temperature type of sauna characterized by fine drops of warm water unlike the high-temperature Finnish sauna characterized by low humidity.

Unfortunately, the effect of mist sauna bathing on human physiological functions has not yet been compared to those of other bathing methods until now. Hence, we investigated the physiological effects of bathing methods including the mist sauna on recovery from muscle fatigue.

## Methods

### Subjects

Ten healthy male students (22 ± 1.2 years, 172 ± 6.20 cm, 62 ± 8.6 kg) participated in this study. Participants were instructed to refrain from any intense muscle activity on the day prior to the experiment. They were asked to refrain from drinking caffeinated beverages, and smoking cigarettes during the 2 h period immediately preceding the experiment. Informed consent for participation in the study, approved by the bioethics committee of the Graduate School of Engineering, Chiba University, was obtained from all subjects.

### Procedure

**Figure 1:**
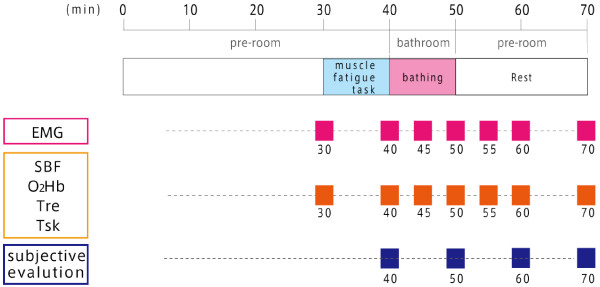
The protocol of the experiment.

The experiment was conducted from May to July 2010. The air temperature and relative humidity of the pre-room were controlled at 27°C and 50%, respectively. In this study, four different bating methods were investigated: full immersion bath, showering, mist sauna, and no bathing as the control. For each condition, the subjects were controlled as follows. In the full immersion bath condition the subject was immersed in hot water (40°C) to the neck. In the mist sauna condition the subject was splashed with mist of fine drops of warm water (40°C). During showering the subjects were drenched in a sitting position outside the bathtub. Four experiments were conducted at the same time of day on separate days. The protocol consisted of four experimental sessions on four different days which were 1 week apart from session to session. The order of the four conditions was counterbalanced between the subjects. They were directed to wear only swimming trunks. After the subjects entered the pre-room, they were asked to relax for at least 30 min. Each subject conducted the muscle fatigue task for 10 min in the pre-room and bathed for 10 min. Then, subjects rested for 20 min in the pre-room. The procedure of the experiment is shown in Figure 1.

### Task

#### Maximal voluntary contraction (%)

A maximal voluntary isometric elbow flexion of the right arm was performed three times during 5 s. The maximum value of the rest trial was taken as the maximal voluntary contraction (MVC) reference value for that individual. To exclude the influence of cumulative fatigue, we measured MVC two days before the experiments.

#### The muscle fatigue-inducing task and the rest contraction task

Each subject was asked to sit down on the chair. To adjust to a fixed posture and elbow level against varying height of the subjects, scaffolding was furnished. In the muscle fatigue-inducing task, targets were set on the visual feedback display at 30% of the MVC reference value (30% MVC). The subject was required to maintain a constant 30% MVC level with constant posture of the elbow flexion (90°) as long as he could maintain the contraction level and the posture. In the test contraction task, to elevate fatigue recovery in the electromyogram (EMG), the subjects performed test contractions of 10% MVC for 15 s in the bathtub.

### Measurements

Rectal temperature (Tre) and skin temperature (Tsk) at four sites (thigh, foot, forearm, chest) were measured with thermistors. The thermistor probe for Tre was inserted 10 cm beyond the anal sphincter. These temperature data were recorded every 2 s by a data logger (LT-8; Gram Corporation). Mean skin temperature (Tsk) was calculated using the formula of Ramanathan [20]. Surface EMG signals were recorded using surface electrodes (TSD150, BIOPAC Systems) which were placed on the right biceps brachii abraded etanol-cleaned skin.

Near-infrared spectroscopy (NIRS) was performed with a NIRO-300 (Hamamatsu Photonics, Japan) at a wavelength of approximately 700 to 1000 nm with probes placed on the right biceps brachii and monitored continuously. Near infrared spectroscopy (NIRS) is a non-invasive diagnostic tool that facilitates the direct monitoring of oxygen saturation and changes in oxyhemoglobin (O2Hb), deoxyhemoglobin (HHb), and total hemoglobin (tHb) concentration in tissues such as the brain and muscle. NIRS determines hemoglobin concentration and saturation, allowing us to determine changes in tissue oxygenation and blood volume from detecting fluctuations in concentration of O2Hb, HHb, and tHb. NIRS offers unambiguous quantification (by separating absorption from scattering) of tissue oxygenation and provides accurate and immediate information on tissue [21,22]. We used O2Hb as the index of muscle blood flow.

The skin blood flow on the forearm (ventral) was measured by a laser Doppler flowmeter (ALF21, Advance Co., Ltd.). Subjects were instructed to keep their arm as stable as possible to minimize artifacts due to the movement of the laser Doppler probe. All signals were converted from analog to digital at a 1 kHz sampling rate (A/D converter: BIOPAC MP150), and were stored in a computer (Dynabook EX/56 L, Toshiba). The subjective evaluation of fatigue was measured by the VAS method.

### Statistical analysis

Changes (△) of the physiological parameters were calculated by subtracting from the value of the muscle fatigue-inducing task immediately after. In the MNF, the change rate (◇) was calculated by dividing with the value of the muscle fatigue-inducing task immediately after. For the physiological parameter, a two-way repeated measures ANOVA (bathing method factor × time factor) was conducted. When a significant F value was found, we performed a Bonferroni as a post-hoc test. In addition, Pearson’s correlation was used to examine the relationship among the bathroom temperature and Tre in the mist sauna condition as well as among the MNF of the EMG and Tre. All statistical analyses were performed using SPSS 18.0 J (SPSS, Japan). The probability level of 0.05 was taken as indicative of statistical significance. Data are shown as mean ± SD of the mean unless otherwise stated.

## Results

The changing rate of MNF (◇MNF) showed a significant main effect of time but not of bathing method (F (3,27) = 0.368, *P* = 0.776). Subsequently, to examine the difference of the bathing method at each time, we conducted a one-way repeated measures ANOVA for bathing method factor. As a result, ◇MNF of the full immersion bath condition was significantly higher than that in the no bathing condition at 70 min (F (4,36) = 7.337, *P* = 0.008 (Table 1).

**Table 1:** The results of the in the ◇MNF

**Time**	**(min)**	**Bathing methods**			
		**Control**	**Showering**	**Mist sauna**	**Full immersion bath**
	0	1 ± 0	1 ± 0	1 ± 0	1 ± 0
Bathing	5	1.063 ± 0.053	1.018 ± 0.019	1.057 ± 0.062	1.049 ± 0.051
	10	1.087 ± 0.084	1.077 ± 0.043	1.089 ± 0.067	1.067 ± 0.053
	15	1.090 ± 0.085	1.090 ± 0.054	1.111 ± 0.083	1.107 ± 0.060
	20	1.096 ± 0.087	1.103 ± 0.065	1.117 ± 0.087	1.134 ± 0.069
	30	1.091 ± 0.091	1.113 ± 0.065	1.136 ± 0.122	1.144 ± 0.076^a^

^a^*P* <0.05 Full immersion bath *vs.* control.

0 to 10 min: Bathing period.

Table 2 shows the results of △Tre. The △Tre showed a significant main effect of bathing method but not of time (F (3,27) = 5.5984, *P* = 0.003). △Tre of the full immersion bath condition was significantly higher than those in the no bathing and showering conditions at 55 min and 60 min. Furthermore, △Tre of the mist sauna condition was significantly higher than those in the showering condition at 55 min.

**Table 2:** The results of ΔTre

**Time (min)**	**Bathing methods**			
	**Control**	**Showering**	**Mist sauna**	**Full immersion bath**
0	0 ± 0	0 ± 0	0 ± 0	0 ± 0
5	−0.012 ± 0.029	0.030 ± 0.071	0.072 ± 0.146	0.122 ± 0.115
10	−0.041 ± 0.030	0.019 ± 0.064	0.144 ± 0.216	0.241 ± 0.203
15	−0.051 ± 0.042	0.015 ± 0.079^a^	0.214 ± 0.277^b^	0.343 ± 0.222^c^
20	−0.078 ± 0.065	0.059 ± 0.102^a^	0.165 ± 0.326	0.318 ± 0.177^c^
30	0.018 ± 0.318	0.026 ± 0.110	0.036 ± 0.385	0.066 ± 0.101

^a^*P* <0.05 Full immersion bath *vs.* showering.

^b^*P* <0.05 Mist sauna *vs.* showering.

^c^*P* <0.05 Full immersion bath *vs.* control.

0 to 10 min: Bathing period.

The △Tsk showed significant main effects for bathing method (F (3.27) = 18.545, *P* = 0.000) and time. The △Tsk of the full immersion bath and mist sauna were significantly higher than those in the showering and no bathing conditions at 45 min and 50 min. In addition, △Tsk of mist sauna condition was higher than that of no bathing condition at 50 min and 55 min (Table 3).

**Table 3:** The results of △Tsk

**Time**	**(min)**	**Bathing methods**			
		**Control**	**Showering**	**Mist sauna**	**Full immersion bath**
	0	0 ± 0	0 ± 0	0 ± 0	0 ± 0
Bathing	5	0.153 ± 0.246	1.378 ± 0.800^a^	2.680 ± 1.581^bc^	3.577 ± 1.367^d^
	10	0.107 ± 0.188^e^	1.501 ± 1.260^a^	3.588 ± 1.656^bc^	3.707 ± 1.482^d^
	15	0.083 ± 0.212^e^	0.756 ± 1.111	1.724 ± 1.352	1.343 ± 0.871
	20	0.053 ± 0.317	0.178 ± 0.841	0.809 ± 0.790	0.829 ± 0.607
	30	0.040 ± 0.328	−0.362 ± 0.824	0.224 ± 0.961	0.466 ± 0.412

^a^*P* <0.05 Full immersion bath *vs.* showering.

^b^*P* <0.05 Mist sauna *vs* showering.

^c^*P* <0.05 Full immersion bath *vs* mist sauna.

^d^*P* <0.05 Full immersion bath *vs* control.

^e^*P* <0.05 Mist sauna *vs.* control.

0 to 10 min: Bathing period.

△O2Hb concentration showed significant main effects ware significant for bathing method (F (3,27) = 12.864, *P* = 0.000) and time (Table 4). Values in the full immersion bath and mist sauna were significantly higher than those in the showering and no bathing conditions from 55 min to 70 min. During the full immersion bath was significantly higher than those during the mist sauna condition.

**Table 4:** The results of O2Hb

**Time (min)**	**Bathing methods**			
	**Control**	**Showering**	**Mist sauna**	**Full immersion bath**
0	0 ± 0	0 ± 0	0 ± 0	0 ± 0
5	12.596 ± 4.638	13.511 ± 2.418	11.683 ± 5.624	13.513 ± 2.301
10	13.994 ± 2.475	14.048 ± 1.662	14.281 ± 2.747	14.056 ± 2.590
15	14.777 ± 2.144^a^	15.076 ± 1.735^b^	21.763 ± 6.825^cd^	23.145 ± 4.413^e^
20	14.159 ± 2.275^a^	15.358 ± 2.961^b^	23.038 ± 3.786^cd^	23.850 ± 4.450^e^
30	14.024 ± 1.892^a^	16.247 ± 2.572^b^	23.438 ± 3.848^cd^	25.117 ± 4.774^e^

^a^*P* <0.05 Mist sauna *vs.* control.

^b^*P* <0.05 Full immersion bath *vs.* showering.

^c^*P* <0.05 Mist sauna *vs.* showering.

^d^*P* <0.05 Full immersion bath *vs.* mist sauna.

e*P* <0.05 Full immersion bath *vs.* control.

0 to 10 min: Bathing period.

Furthermore, △SBF showed significant main effects for bathing method (F (3,27) = 35.909, *P* = 0.000) and time. Values in the full immersion bath and mist sauna were significantly higher than those in the showering and no bathing conditions from 45 min to 70 min (Table 5). The full immersion bath was significantly higher than those during the mist sauna condition.

**Table 5:** The results of △SBF

**Time (min)**	**Bathing methods**			
	**Control**	**Showering**	**Mist sauna**	**Full immersion bath**
0	1 ± 0	1 ± 0	1 ± 0	1 ± 0
5	−0.387 ± 0.815^ab^	1.278 ± 1.186^c^	5.784 ± 3.121^d^	6.258 ± 2.789^e^
10	−0.349 ± 0.770^ab^	0.132 ± 1.040^c^	6.875 ± 3.196^d^	7.257 ± 3.381^e^
15	−0.088 ± 1.009^ab^	−0.231 ± 0.909^c^	3.776 ± 1.614^d^	4.760 ± 2.812^e^
20	−0.302 ± 1.082^ab^	−0.403 ± 0.653^c^	2.707 ± 2.197^d^	2.868 ± 1.970^e^
30	−0.574 ± 0.561^ab^	−0.403 ± 0.653^c^	1.440 ± 1.278^d^	2.029 ± 2.027^e^

^a^*P* <0.05 Full immersion bath vs. showering. ^b^*P* <0.05 Mist sauna *vs.* control.

^c^*P* <0.05 Mist sauna vs. showering. ^d^*P* <0.05 Full immersion bath *vs.* mist sauna.

^e^*P* <0.05 Full immersion bath *vs.* control.

0 to 10 min: Bathing period.

**Figure 2:**
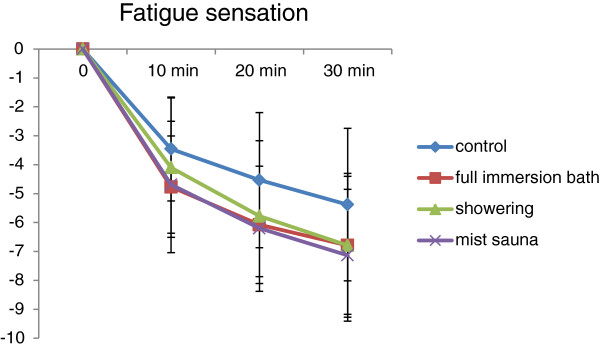
The result of the fatigue sensation.

On the other hand, in the fatigue sensations, the main effect of time factor was significant but that of the bathing method factor was not significant (F (3,27) = 4.052, *P* = 0 .017) (Figure 2).

**Figure 3:**
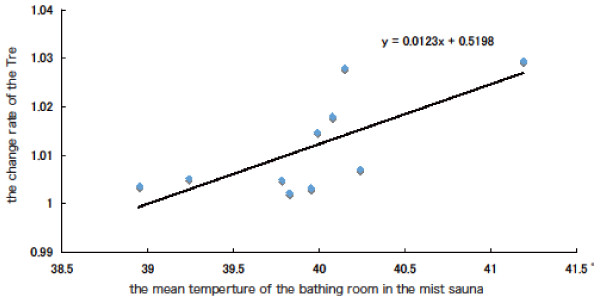
The relationship between △Tre and the average of the bathroom temperature during the mist sauna condition.

**Figure 4:**
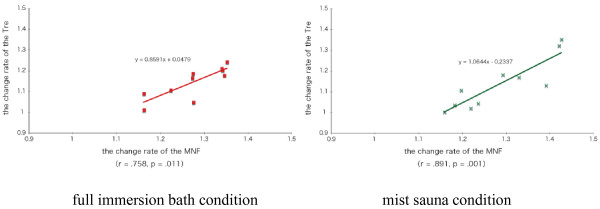
The relationship between △Tre and △MNF during the full immersion bath and mist sauna conditions.

Figure 3 shows the relationship between ◇Tre and the average of the bathroom temperature during the mist sauna condition in each subject. ◇Tre was quite correlated with the average of the bathroom temperature (r = 0.683, *P* = 0.042). Figure 4 shows the relationship between ◇MNF of EMG and ◇Tre in full immersion bath and mist sauna. ◇MNF was correlated with ◇Tre in the full immersion bath condition (r = 0.758, *P* = 0.011) and mist sauna condition (r = 0.891, *P* = 0.001).

## Discussion

This is the detailed study on the physiological effects of bathing methods including the mist sauna on recovery from muscle fatigue. We investigated the physiological effects of bathing methods on recovery from muscle fatigue using the EMG, Tre, Tsk, O2Hb, SBF, et cetera.

In this experiments, we found that ◇MNF of EMG during the full immersion bath condition was significantly higher than that during the no bathing condition. This means that the subjects made a good recovery from muscle fatigue during the full immersion bath.

The △Tre of the full immersion bath condition was significantly higher than those in the no bathing and showering conditions. From these results, we obtained that the full immersion bath was effective on recovery from muscle fatigue compared to showering and no bathing.

In the △SBF and △O2Hb, during the full immersion bath was significantly higher than those during the mist sauna condition. We considered that there are the effects of physiological responses by the presence or absence of water pressure as one reason. From these results of the blood flow, maybe it was estimated that there is the effect of water pressure in the full immersion bath and there is little influence of the water pressure in the mist sauna condition. It was presumed that blood flow enhanced by the increased venous return.

In addition, the △Tsk during the full immersion bath and mist sauna were significantly higher than those during showering and no bathing conditions at 50 min. From these results, the mist sauna was nearly equal to the full immersion bath in effectiveness for recovery from muscle fatigue. Furthermore, it was shown that △Tsk of the mist sauna condition was significantly higher than that of other conditions at 55 min (5 min after bathing).

By contrast, the ◇MNF of EMG during the mist sauna was not significantly higher than that during the other conditions. We speculated that this lack of difference might be associated with individual differences of ◇Tre. In the mist sauna, Tre values of half the subjects were not higher (1.01°C below); on the other hand, that of the resting subjects increased. The bathroom temperature of the former case was 40°C or below, and that of the latter case was more than 40°C. Therefore, we confirmed that the standard deviation (SD) of Tre were a bit larger during the mist sauna condition (SD = 0.23°C) than that during the showering (SD = 0.17°C) and full immersion bath (SD = 0.19°C). In the mist sauna condition, the bathroom temperature of half the subjects was lower than the standard setting (40°C) due to the subjects’ movements in and out between bathroom and pre-room. Presumably, some of the subjects took more time to move between rooms than others. We considered whether the individual difference of the bathroom temperature in the mist sauna condition affected recovery from muscle fatigue. To examine these relationships, we calculated two correlations, bathroom temperature and ◇Tre and the ◇MNF of EMG and ◇Tre in the mist sauna condition. A positive correlation was found between ◇MNF and ◇Tre. This result suggests that the higher bathroom temperature provided the higher ◇MNF. A positive correlation was also found between bathroom temperature and ◇Tre.

In other words, if the rectal temperature of the subject who had low bathroom temperature was high, the Tre of the mist sauna condition was high, and MNF might be higher. As a result, the effects of the mist sauna might be almost same as those of the full immersion bath if the bathroom temperature were held at 40°C. We verified that the thermal sensation of the mist sauna was equal to that of the full immersion bath.

In summary, the present results suggest that both full immersion bath and mist sauna induced recovery from muscle fatigue.

## Abbreviations

MNF: Mean power frequency; EMG: Electromyogram; Tre: Rectal temperature; Tsk: Skin temperature; SBF: Skin blood flow; O2Hb: Concentration of oxygenated hemoglobin.

## Competing interests

The authors declare that they have no competing interests.

## Authors’ contributions

SL wrote the manuscript. SI performed the experiments, analyzed the data. TK conceived and designed the study. SL, SI, TK and YS were responsible for coordination of the study and oversight of data collection and analysis. All authors have read and approved the final manuscript.

## References

[B1] Hayasaka S Shibata Y Noda T Goto Y Ojima T Incidence of symptoms and accidents during baths and showers among the Japanese general public J Epidemiol 2011 21 305 308 10.2188/jea.JE20100136 21478641 PMC3899424

[B2] Traphagan JW Culture and long-term care: the bath as social service in Japan Care Manag J 2004 5 53 60 10.1891/cmaj.5.1.53.61263 15792331

[B3] Hirate K Kamata M Ishiwatari H Kuwasawa Y Ishikawa N Iio A Asano Y Kiya F Bougaki K Tanaka M Study on design criteria for domestic hot water supply system by the investigation with questionnaire. Part 2 Investigation of bathroom and dressing room, and required standards in using hot water JSHASE Trans 1993 52 71 80 [In Japanese with English abstract]

[B4] Hayasaka S Shibata Y Goto Y Noda T Ojima T Bathing in a bathtub and health status: a cross-sectional study Complement Ther Clin Pract 2010 16 219 221 10.1016/j.ctcp.2010.05.002 20920807

[B5] Onaka T Tochihara Y Kubo M Yamaguchi C Physiological and subjective responses to standing showers, sitting showers, and sink baths Appl Human Sci 1995 14 235 239 10.2114/ahs.14.235 8528937

[B6] Chaudhuri A Behan PO Fatigue in neurological disorders Lancet 2004 363 978 988 10.1016/S0140-6736(04)15794-2 15043967

[B7] Grandjean E Fatigue Am Ind Hyg Assoc J 1970 31 401 411 10.1080/0002889708506267 5473750

[B8] Ream E Richardson A Fatigue: a concept analysis Int J Nurs Stud 1996 33 519 529 10.1016/0020-7489(96)00004-1 8886902

[B9] Ream E Richardson A Fatigue in patients with cancer and chronic obstructive airways disease: a phenomenological enquiry Int J Nurs Stud 1997 34 44 53 10.1016/S0020-7489(96)00032-6 9055120

[B10] Mizuno K Tanaka M Tajima K Okada N Rokushima K Watanabe Y Effects of mild-stream bathing on recovery from mental fatigue Med Sci Monit 2010 16 CR8 CR14 20037494

[B11] Johnston BL Watt EW Fletcher GF Oxygen consumption and hemodynamic and electrocardiographic responses to bathing in recent post-myocardial infraction patients Heart Lung 1981 10 666 671 6909192

[B12] Bennet AF Thermal dependence of muscle functions Am J Physiol Regulatory Integrative Comp Physiol 1984 247 R217 R229 10.1152/ajpregu.1984.247.2.R217 6380314

[B13] De Ruiter CJ Jones DA Sargeant AJ De Hann A Temperature effect on the rates of isometric force development and relaxation in the fresh and fatigued human adductor pollicis muscle Exp Physiol 1999 84 1137 1150 10.1017/S0958067099018953 10564710

[B14] Rall JA Woledge RC Influence of temperature on mechanics and energetics of muscle contraction Am J Physiol Regulatory Integrative Comp Physiol 1990 259 R197 R203 10.1152/ajpregu.1990.259.2.R197 2201213

[B15] Ranatunga KW Temperature dependence of shortening velocity and rate of isometric tension development in rat skeletal muscle J Physiol (Lond) 1982 329 465 483 7143257 10.1113/jphysiol.1982.sp014314 PMC1224791

[B16] Davies CTM Young K Effect of temperature on the contractile properties and muscle power of triceps surea in humans J Appl Phyisiol 1983 55 191 195 10.1152/jappl.1983.55.1.191 6885568

[B17] De Ruiter CJ De Hann A Temperature effect on the force/velocity relationship of the fresh and fatigued human abductor pollicis muscle Pflugers Arch 2000 440 163 170 10864011 10.1007/s004240000284

[B18] Ranatunga KW Temperature dependence of mechanical power output in mammalian (rat) skeletal muscle Exp Phyiol 1998 83 371 376 10.1113/expphysiol.1998.sp004120 9639346

[B19] Sargeant AJ Effect of muscle temperature on leg extension force and short-term power output in humans Eur J Appl Phyiol 1987 56 693 698 10.1007/BF00424812 3678224

[B20] Ramanathan NA A new weighting system for mean surface temperature of the human body J Appl Physiol 1964 19 531 533 14173555 10.1152/jappl.1964.19.3.531

[B21] Hausser-Hauw C Rakotonanahary D Fleury B Obstructive-sleep apnea syndrome, brain oxygenation measured with near-infrared spectroscopy Neurophysiol Clin 2000 30 113 118 10.1016/S0987-7053(00)00063-0 10812580

[B22] Jenni O Wolf M Hengartner M Siebenthal K Keel M Bucher HU Impact of central, obstructive and mixed apnea on cerebral hemodynamics in preterm infants Biol Neonate 1996 70 91 100 10.1159/000244353 8864428

